# Deep Convolutional Neural Networks for Detecting COVID-19 Using Medical Images: A Survey

**DOI:** 10.1007/s00354-023-00213-6

**Published:** 2023-04-04

**Authors:** Rana Khattab, Islam R. Abdelmaksoud, Samir Abdelrazek

**Affiliations:** Information Systems Department, Faculty of Computers and Information, Mansoura University, Mansoura, Egypt

**Keywords:** Artificial intelligence, COVID-19, Imaging modalities, Deep learning

## Abstract

Coronavirus Disease 2019 (COVID-19), which is caused by Severe Acute Respiratory Syndrome Coronavirus 2 (SARS-COV-2), surprised the world in December 2019 and has threatened the lives of millions of people. Countries all over the world closed worship places and shops, prevented gatherings, and implemented curfews to stand against the spread of COVID-19. Deep Learning (DL) and Artificial Intelligence (AI) can have a great role in detecting and fighting this disease. Deep learning can be used to detect COVID-19 symptoms and signs from different imaging modalities, such as X-Ray, Computed Tomography (CT), and Ultrasound Images (US). This could help in identifying COVID-19 cases as a first step to curing them. In this paper, we reviewed the research studies conducted from January 2020 to September 2022 about deep learning models that were used in COVID-19 detection. This paper clarified the three most common imaging modalities (X-Ray, CT, and US) in addition to the DL approaches that are used in this detection and compared these approaches. This paper also provided the future directions of this field to fight COVID-19 disease.

## Introduction

**Fig. 1:**
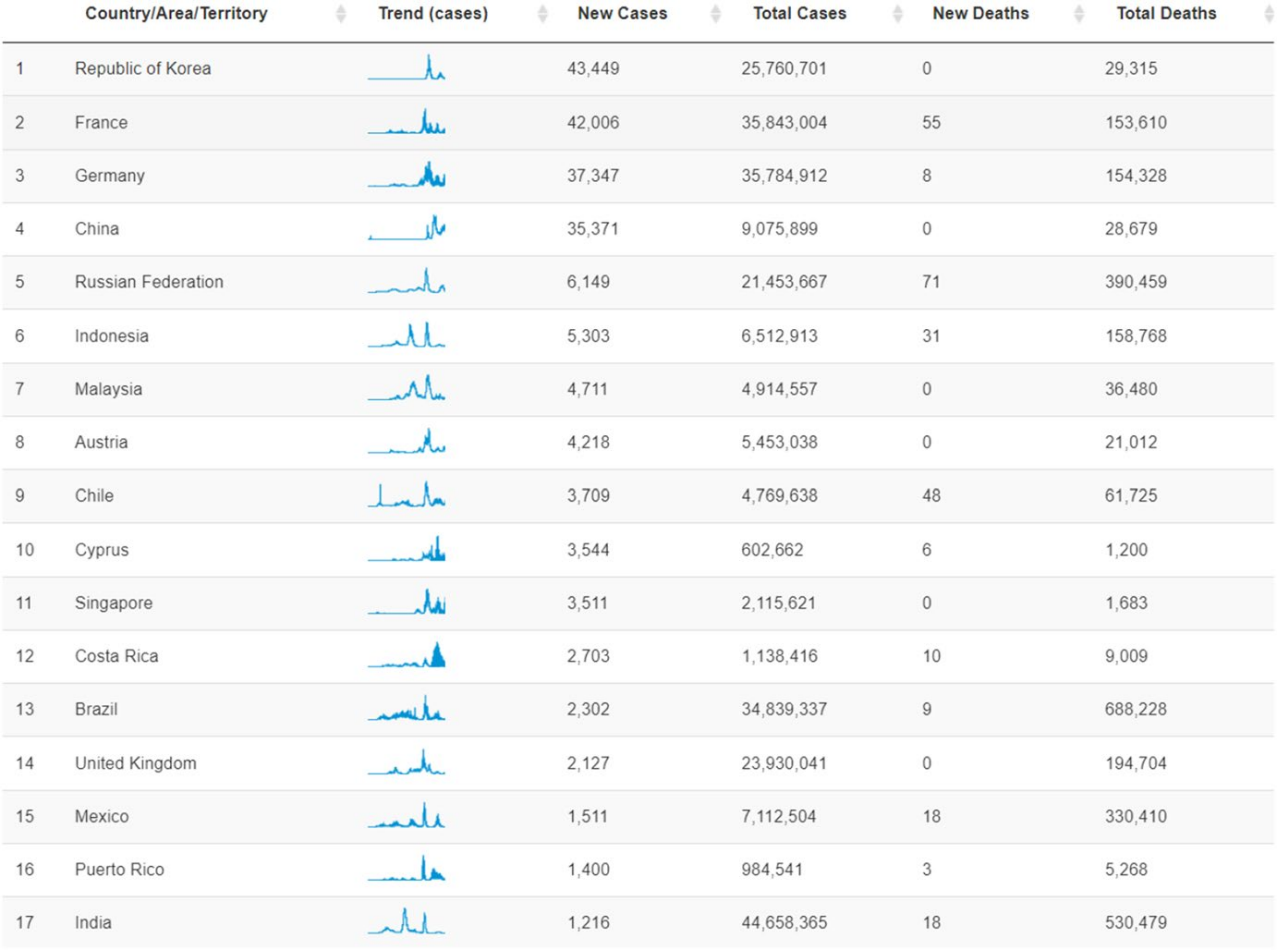
The total COVID-19 cases and deaths for most affected countries from January 2020 until November 2022

Recently, people all over the world have heard and known about COVID-19. Health authorities in China notified the World Health Organization (WHO) which is an agency of the united nations that its goal is promoting the health [1]. on December 8, 2019, about different cases of a novel virus that affects the respiratory system [2]. After 1 month, on January 7, 2020, WHO declared that the 2019 Novel Coronavirus ( 2019-NCOV) is an abbreviation for the novel coronavirus pandemic [3]. Then, a coronavirus research group termed it SARS-COV-2 [4]. In late January 2020, it was re-titled COVID-19 by WHO as an abbreviation for Coronavirus Disease 2019. Finally, on March 13, 2020, WHO proclaimed COVID-19 a global pandemic [5]. COVID-19 continued in its great spread and affected more countries. China informed that there were 12,000 suspected COVID-19 cases and about 7736 positive COVID-19 cases on January 30, 2020. On the same day, various suspected cases appeared in 18 countries [6]. Meanwhile, in the year 2021, these cases have increased. On March 18, 2021, the United States announced that it had 29,260,772 COVID-19 positive cases. COVID-19 continued in its spread in 2021 and 2022. Figure 1 shows the total COVID-19 cases and deaths for the most affected countries from January 2020 until November 2022 [7].

This survey contributions are highlighted in:

1. Providing a comprehensive study about COVID-19 sources, COVID-19 symptoms, Coronaviruses’ families and their subgroups, and how the virus has been transmitted to humans as shown in Sect. 2.
2. Discussing COVID-19 detection from different ways such as blood tests, viral tests, and imaging modalities; highlighting the main differences among them as shown in Sect. 2.2.
3. Providing a comparative study about COVID-19 detection through different image types: X-Ray, CT, Ultrasound. In addition to multi-modal-based detection; illustrating the main features, advantages, and disadvantages of each modality, as highlighted in Sects. 2.2 and 7.
4. Providing a comparative comparison study among more than 100 scientific papers for COVID-19 detection based on their imaging modalities, the employed techniques, datasets, limitations, evaluation measures, publication dates, and publication sources, as shown in Tables 3, 4, 5, and 7.
5. Discussing COVID-19 detection using deep learning techniques; highlighting the main deep learning architectures and their characteristics, as shown in Sect. 4. Also, highlighting the main advantages and limitations of the different deep learning models and how to overcome these limitations, as shown in  Sect. 9.
6. Discussing the most frequently used COVID-19 datasets and providing a detailed description about them, as shown in Table 8, and Sect. 8

## Sources of Coronaviruses’ Families and Their Subgroups

**Fig. 2:**
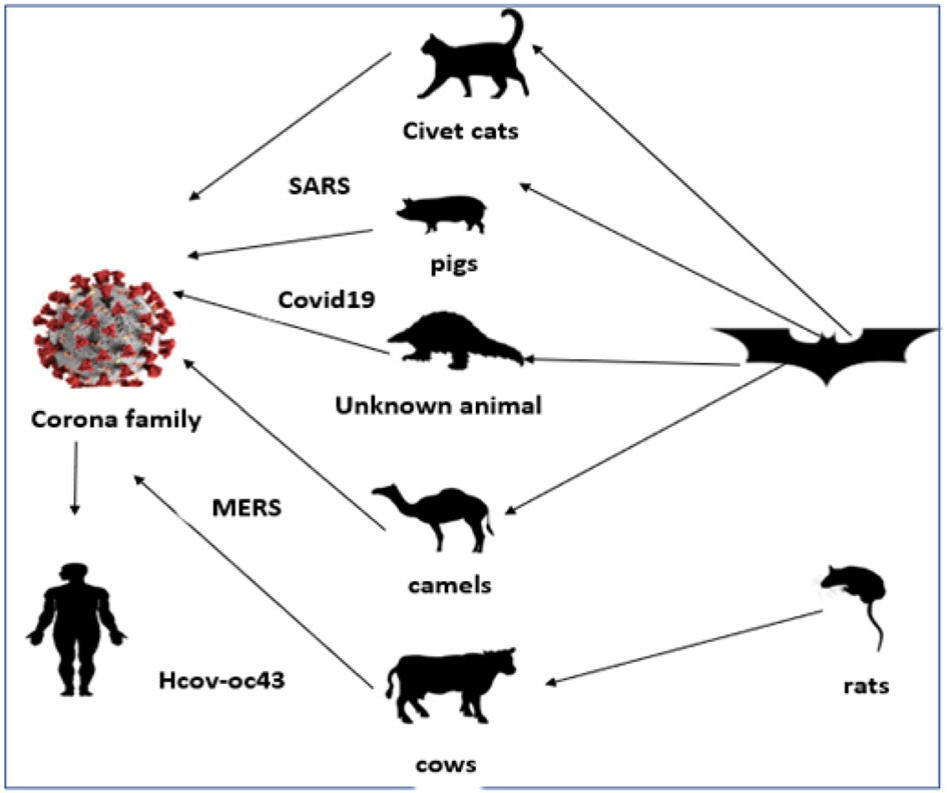
Coronaviruses’ families’ transmission to humans

Coronaviruses are classified into four families: alpha $\alpha$, beta $\beta$, gamma $\gamma$, and delta $\delta$. Beta $\beta$ group contains Severe Acute Respiratory Syndrome (SARS-COV and SARS-COV-2) [8]. About 8000 confirmed cases with coronaviruses, especially SARS-COV, existed between 2002 and 2003. In 2012 WHO reported 2494 positive cases of the Middle East Respiratory Syndrome (MERS). After various studies, it was found that MERS came from Arabian camels [9, 10]. Studies also showed that SARS-COV-2 infected 750,000 cases in 150 countries yielding a 4% death rate. However, the death rate of SARS-COV was 9%, affecting 26 countries. From these studies, it can be concluded that the real danger of the coronaviruses’ families, especially COVID-19, is its rapid spread. This is due to a specific genetic event in SARS-COV-2 Spike protein’s Receptor-Binding Domain (RBD) [11], where RBD protein closely binds Human and bat Angiotensin-Converting Enzyme 2 (ACE2) receptors [12]. As shown in Fig. 2, the coronaviruses’ families are transmitted to humans from bats and other wild animals after some changes in their genetic structures, which could threaten humans’ lives [13, 14].

### COVID-19 Symptoms and Signs

COVID-19 infection has high spread rates among humans. The cause of these great spread rates is unknown. The symptoms of COVID-19 include difficulty in breathing, fever, and severe cough [15]. WHO reported other symptoms, such as chest discomfort and bilateral infiltration of lungs [16–18]. Some symptoms, such as discoloration of toes or fingers and rash on the skin, do not appear in children and adults. Older people and people infected with chronic diseases which are long-term human health conditions or diseases, especially a sickness that develops over time [19], are more prone to acquire COVID-19 infected, as a result of which they loss their lives [18]. Some studies showed that children below 10 years old have a lower chance of getting infected or passed away by COVID-19 [20]. Until April 2020, only one case of adults passed away by COVID-19 [21–23]. Until February 2020, it was reported that only one baby had a severe kind of virus [24]. Studies showed that children could catch this virus if they are in contact with people infected with COVID-19 [25].

### COVID-19 Detection

**Fig. 3:**
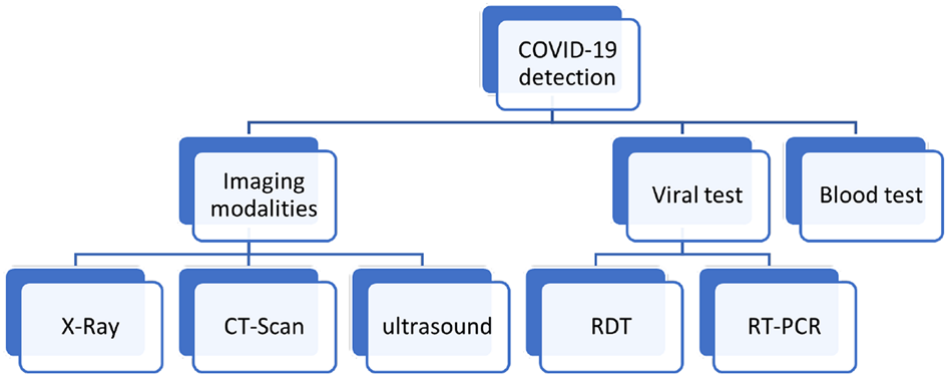
COVID-19 detection approaches

Fighting COVID-19 is not an easy task, as it might be thought since the virus has a rapid spread rate among citizens across all countries around the world. Moreover, it can be developed by itself and make another strain. Therefore, early detection of COVID-19 is the true weapon to beat it. Figure 3 shows that this detection can be achieved through three main approaches. These approaches are either a blood test, viral test, or analysis of different imaging modalities, such as X-ray, Computed Tomography (CT) scan, and Ultrasound (US) [26]

**Fig. 4:**
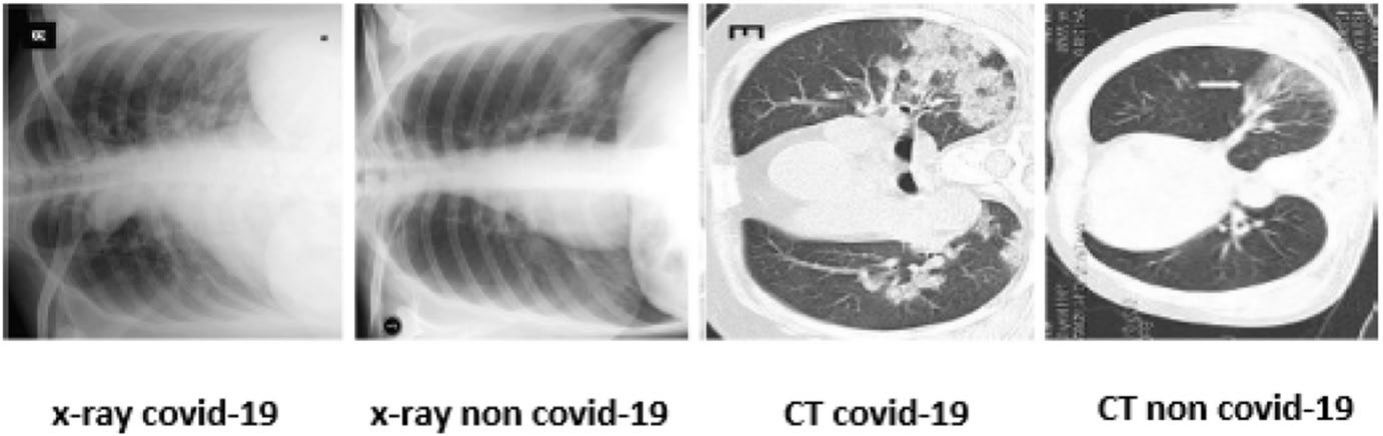
Main features of COVID-19 for both CT and X-ray images

The blood test is used to detect the existence of antibodies for SARS-COV-2. On the other hand, blood analysis sensitivity for detecting SARS-COV-2 ranges from 2 to 3% [27]. The viral test has two approaches: rapid diagnostic test (RDT) and reverse transcription-polymerase chain reaction (RT-PCR). RDT is used in antibodies detection, and it can give a quick result in about half an hour. However, it is not recommended for COVID-19 detection as its accuracy depends on the quality of the sample, and It is unable to classify COVID-19 and other viral pneumonia [28] [29]. Where viral pneumonia is a contagious lung syndrome produced by an virus such as influenza [30]. RT-PCR is regarded as the truly accurate approach for COVID-19 detection [31]. However, it has some limitations. It is an expensive method and time-consuming approach. Moreover, it has lesser sensitivity in COVID detection than imaging modalities [32], as its sensitivity ranges from 50 to 62% [33]. Detecting COVID-19 through imaging is the best way to obtain rapid and accurate results. X-ray images have many advantages that encourage researchers to use them for COVID-19 detection. These advantages include its lower cost than other imaging modalities and its huge availability. Moreover, the amount of radiation during acquiring X-ray images is less than that of CT scan images. Therefore, it is used in detecting different diseases, such as lung cancer and cardiac diseases. The use of X-ray images has significantly spread in many places, especially in poor countries [34]. However, CT scan images have higher quality than X-ray images [35]. Therefore, CT scan images have more accurate diagnosis results. However, CT scan images have some disadvantages, such as their high cost and patients being exposed to more radiation. X-ray and CT scan images have popular features for COVID-19 identification. X-ray images use features, such as ground-glass opacification in the higher right section of the lung. However, CT scan images use features represented by ground-glass areas in the lower side of the lung and halo sign and consolidation areas in lower lobes [36–40]. Figure 4 compares COVID-19 cases to non-COVID cases for both X-ray and CT imaging features [41].

## Methodology

The detection of COVID-19 either from X-Ray, CT, or US was reviewed using a variety of DL architectures, particularly CNNs. In this study, we presented a comprehensive analysis of the researches that have been cited. The survey adopted keele [42] and [43] methodology for formulating a systematic literature review to illustrate research questions, search strategy, and the used datasets and AI tools in building predictive COVID-19 detection models.

### Research Questions

COVID-19 has emerged as a global issue that required attention. Therefore, AI researchers proposed multiple models for accurate detection of COVID-19. In this study, the following major question was posed:

**RQ** What role did AI play in the development of accurate models for COVID-19 detection ?

The subsequent sub-questions were asked to answer this question:

**Sub-RQ1** What are the main approaches for COVID-19 detection?

**Sub-RQ2**Which imaging modalities give more accurate results? And what are advantages and disadvantages of each modality?

**Sub-RQ3** How can COVID-19 be detected using AI and what AI tools are used in this detection?

**Sub-RQ4** What are software tools and datasets used in building predictive COVID-19 detection model?

**Sub-RQ5** How can DL provide a great weapon for fighting COVID-19 and what are the challenges it faced?

### Search Strategy

When conducting the literature search for this study, researchers took into account studies that tackled COVID-19 automatic detection from a variety of angles. The four steps of the search technique were as follows: identifying the information’s sources, creating the search formula, choosing the most relevant primary research, and quality assessment.

#### Identifying the Information’s Sources

Finding and selecting the information sources that would be used to conduct the systematic review was the first step of the search strategy. We searched several digital libraries to find pertinent study publications, such as: Google Scholar (https://scholar.google.com/), Pubmed (https://pubmed.ncbi.nlm.nih.gov/), ResearchGate (https://www.researchgate.net/), IEEE Explore (https://ieeexplore.ieee.org/Xplore/home.jsp), and Springer Link (https://link.springer.com/).

#### Creating the Search Formula

To find the primary studies, a search string that reads as follows was defined: ((“CAD System*”) OR ((“deep learning*”) OR ((“artificial intelligence*”) OR (“imaging modality*”) AND (“COVID-19 detection*”)). This string was solely used for document titles. The search phrase was changed to work in each of the chosen libraries.

#### Choosing the Most Relevant Primary Research

The study’s inclusion criteria were created to ensure that only ideas that examined and applied AI and image methods to detect COVID-19 were considered. In addition, three exclusion criteria that attempted to weed out papers that didn’t promote the research were defined. Duplicate documents, researches written in a language other than English, and unobtainable documents were the exclusion criteria. These requirements were used to filter out articles that were found using the search keyword.

#### Quality Assessments

All included publications were evaluated for quality based on the research provided in them as well as the exclusion criteria. For this literature study, high-quality papers that covered the application of imaging technology and deep learning to identify COVID-19 were chosen. In order to establish a comprehensive evaluation of the study’s quality, we also developed a quality standard based on the following three factors that influence study quality:

1. Is information about the datasets and their citations included in the study?
2. Is data analysis procedure proper?
3. Did accuracy or any other evaluation measures serve as a gauge of the models’ quality?

## Popular Convolutional Neural Network Architectures for COVID-19 Detection

Deep Learning (DL) algorithms provide better accuracy than classical machine learning algorithms [44]. They can deal with a huge number of data and raw images to extract knowledge and information without the need to enhance or segment these images [45]. DL algorithms also provide better improvements in image analysis [46]. They are used in disease detection, such as COVID-19 and retinal diseases which are affecting the iris and delicate nerve on the flip side of the eye and produce blindness [47]. Additionally, they are used in classification modalities, such as Magnetic Resonance Imaging (MRI) images [48–50]. Where MRI is a medical imaging technology that creates detailed images of body’s organs and tissues by combining a magnetic field with computer-generated radio waves [51]. As shown in the next sections, researchers used DL algorithms, such as Convolutional Neural Network (CNN), which is a procedure of Artificial Neural Network (ANN). CNN is made up of four layers: convolution, pooling, fully linked, and non-linearity. It is an excellent method for improving pattern recognition and images classification performance [52]. To detect novel coronaviruses, some researchers used different CNN architectures, such as Visual Geometry Group (VGG), Residual Convolution Neural Network (ResNet), and Dense Convolution Network (DenseNet). The selection of suitable CNN architectures is based on the size and the nature of the data.

Recently, CNN architectures have accomplished better performance in most complicated tasks, such as medical image analysis and disease detection [26, 53]. In 1998, Yann LeCun designed LeNet as the earliest effective CNN. It was used to detect handwritten digits. It consisted of three convolution layers, two pooling layers, and two fully connected layers [54]. In the next sections, some of the most common CNN architectures are being discussed.

### AlexNet

AlexNet is designed by (Alex Krizhevsky) and is like LeNet. But AlexNet is deeper. It has more filters, stacked convolution layers, dropout, and max pooling. In 2012, AlexNet provided 17% top-five error rate and won the contest of ImageNet Large Scale Visual Recognition Challenge (ILSVRC) [55] which continued every year from 2010 till 2017. Most of researchers use AlexNet in COVID-19 detection to overcome the problem of overfitting, which happens when DL models achieve better accuracy for training data than testing data.

### GoogLeNet

GoogLeNet is deeper than AlexNet, it contains 22 layers and 27 layers, if pooling layers are taken into account. In 2014, GoogLeNet won ILSVRC contest and achieved 6.67% top -five error rate. An inception module (IM) is a major component in GoogleNet. This module functions as a tiny network and can learn both spatial and cross-channel correlations (depth-wise). The IM has various benefits such as, allowing the training of models that are considerably deeper while having ten times less learnable parameters. The number of feature maps in an IM’s output is configured to be less than its input, this reduces the dimensionality of the IM. In addition to the spatial and depth dimensions, an IM is capable of capturing complicated patterns at various scales. [56].

### VGGNet

VGGNet achieved 7.3% top-five error rate in ILSVRC contest in 2014. It contains 19 convolution layers. VGGNet is simpler than AlexNet, it has three fully connected layers. Therefore, it is used in many fields. VGGNet was developed at Oxford university by Visual Geometry Group [57]. The architectural simplicity of VGGNet is a benefit. Nevertheless, it used three times as many parameters as AlexNet. Advanced object identification models are built on the VGG architecture. The VGGNet, which was created as a deep neural network, performs better than baselines on a variety of tasks and datasets outside of ImageNet. In addition, it remains one of the most widely used image recognition architectures today.

### ResNet

ResNet has a residual module that contains a standard layer and a skip connection. ResNet won ILSVRC contest in 2015 with containing 152 layers and provided 3.6% top-five error rate. By linking the input signal of a layer to its output, the skip connection enables that layer’s input signal to go across the network. Thus, the Residual Units (RUs) allowed for the training of a model with 152 layers, which is incredibly deep. The skip connection joins layer activations to subsequent layers. Consequently, a block is created. These discarded building blocks are stacked to create ResNets. The benefit of including this kind of skip link is that regularization will skip any layer that degrades architecture performance. By doing so, an extremely deep neural network can be trained without encountering issues of vanishing or exploding gradients. [58].

### Inception

Inception is an image model block module that seeks to simulate an ideal local sparse structure in a CNN. The Inception network was first created by a team in Google in 2014 with the name Inception V1 in 2014. Inception architecture uses many filters of various sizes on the same level and the idea behind this is to prevent data overfitting from happening and solving computational expense problems. It combines several filter sizes into a single image block rather than being limited to a single filter size, which is then pass to the following layer. [59].

### Xception

Xception is developed by a Google team with depth-wise separable convolutions. The name Xception is derived from extreme Inception, so Xception can be considered as an interpretation of the Inception modules. Entry flow, middle flow, and exit flow are the three structures that make up the Xception architecture. Each of these three topologies is made up of 14 modules (four, eight, and two, respectively), totaling 36 convolution layers. The entry flow, the middle flow, which is repeated eight times, and the exit flow are all the steps that the data must initially go through. Keep in mind that batch normalization comes after convolution and separable convolution layer [59]

### MobileNet

MobileNet is a widely used CNN-based model for image classification. The primary benefit of adopting the MobileNet architecture is that the model requires significantly less computational effort than the traditional CNN model, making it appropriate for use with mobile devices and computers with limited computational power. MobileNet has depth-wise separable convolution layers and ReLU non-linearity, while the final layer is fully connected followed by the SoftMax classification layer. A trade-off between latency and precision is introduced by MobileNet. By using these hyper-parameters, the model builder can select the appropriate model size for their application while taking into account the limitations of the issue [60, 61].

### DenseNet

DenseNet is a kind of convolution neural network that has a top-five error rate of 6.12%, although it uses fewer parameters and costs less to compute than other cutting-edge CNN architectures like ResNet. Through the use of Dense Blocks, which connect all layers directly with one another when their feature-map sizes match, DenseNet makes use of dense connections between layers. In order to maintain the feed-forward nature, each layer receives extra inputs from all earlier layers and transmits its own feature-maps to all later layers. In contrast to the standard CNN architecture, which uses *L* connections between *L* layers, DenseNet uses *L*(*L* + 1)/2-layer connections. The feature-maps of all layers before it is utilized as inputs for each layer, and its own feature-maps are used as inputs into all levels after it. DenseNets offer a variety of appealing benefits, including the elimination of the vanishing-gradient issue, improved feature propagation, promoted feature reuse, and significantly fewer parameters. [62]

## Basic Evaluation Measures Terminologies

In this section, we will review some of scientific terms as well as evaluation measures that are used in evaluating performance in COVID-19 detection. All these measures have equations used in evaluating the classification performance as shown in Table 2. Table 1 shows measures of True positives and True negatives results.

**Accuracy**: This parameter assesses a model’s overall performance. It’s calculated as the model’s proportion of correctly identified data samples [26].

**Recall, Sensitivity or True Positive Rate (TPR)**: This parameter represents the amount of confirmed scenarios that the model properly expected [26].

**Precision**: This metric assesses the model’s ability to predict positive samples with reasonable accuracy [63].

**Specificity**: This metric represents the model’s negative instances [63].

**F1-Score**: a technique for integrating sensitivity and precision into one statistic that take them into account [64].

**Receiver Operating Characteristic (ROC)**: The exchange between precision and sensitivity across a sequence of cut-off points is depicted in this graph. The classifier is better if the curve is close to the upper left corner [26].

**Area Under the Curve (AUC)**: When evaluating a classifier, the AUC of the ROC is employed. The AUC of a perfect classifier would be one [26].

**Table 1:** Measures of true positives and true negatives results

	Diseased	Non-diseased
Test positive	True positives	False positives
Test negative	False negatives	True negatives

**Table 2:** Summary of evaluation measures formulas

Evaluation measure	Formula
Accuracy	(TP+TN)/(TP+TN+FP+FN)
Precision	TP / (TP + FP)
Recall	TP / (TP + FN)
AUC	.5*((TP/TP+FN) +(TN/TN+FP))
F-Measure	(2 * Precision *Recall)/(Precision+ Recall)
Sensitivity	(TP/ total diseased) *100
Specificity	(TN/ total non-diseased) *100
TPR	TP / (TP + FN)
TNR	TN/(TN+FP)

## Data Augmentation and Transfer Learning Terminologies

In this section, we will review some of data augmentation methods that are used for balancing COVID-19 datasets for getting better performance.

**Data augmentation**: A regularization method that uses numerous transformations like flipping, rotating, moving, and resizing o generate a large number of false data samples [26].

**Random Image Cropping and Patching (RICAP)**: RICAP creates a new image by cropping and patching a random number of photographs. As a result, RICAP selects subsets of original features from the images at random and discards the rest, increasing the variety of training images [65].

**Synthetic Minority Oversampling Technique (SMOTE)**: A data augmentation technique that concerns with minority class for making data balance.

**Class-weighted entropy**: It means that When class weighting is turned on, a weighted sum takes place the entire ensuring that every example adds proportionally to the loss. This implies that samples from the smaller classes contribute more to the overall loss [66].

**Cost-sensitive learning**: It’s an imbalanced learning sub-field that deals with classification on datasets with skewed class distributions. When a model is being prepared, prediction errors costs are taken into account (and maybe other expenses) [67].

**ImageNet**: A dataset of 14,197,122 marked photographs of various common items such as creatures, technology, plants and meals, grouped according to WordNet hierarchy [26, 68].

**Transfer learning**: A representation learning idea established on the hypothesis that particular characteristics are familiar to several jobs. By this strategy, a model that has been accomplished in one environment is employed to boost generality in a different situations [69].

## COVID-19 Detection-Related Work

**Fig. 5:**
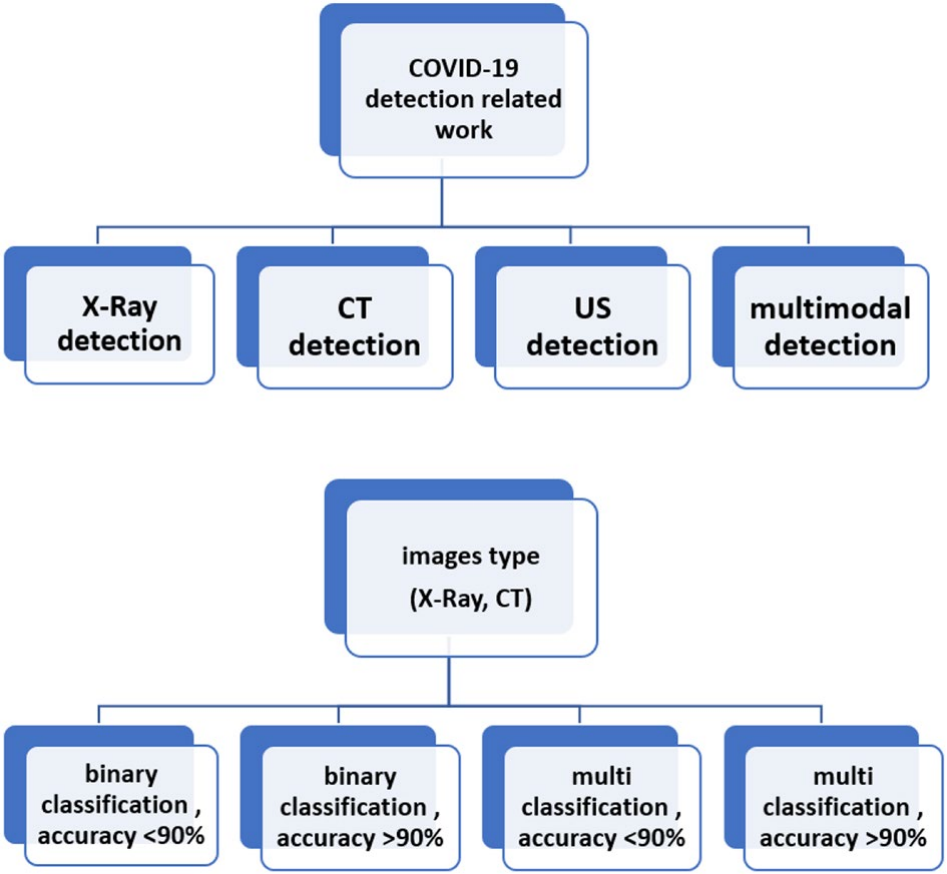
Categorization of related work studies for COVID-19 detection

In this section, literature reviews are provided about COVID-19 detection, either through CT, X-ray, ultrasound images, or through multi-model images by applying AI techniques.The papers included in this literature review are divided into four main categories: COVID-19 detection through X-Ray images, COVID-19 detection through CT images, COVID-19 detection through Ultrasound images, and COVID-19 detection through multi-modal images. Figure 5 illustrates these major categories of the different papers used for COVID-19 detection.

AS shown from Fig. 5, These papers were organized into four categories based on the type of classification and the accuracy percentage: The first two categories are based on whether the papers make a binary classification that yielded to an accuracy more than 90% or binary classification that yielded to an accuracy less than 90%. The other two categories are based on whether the papers make multi-class classification that yielded to an accuracy more than 90% or multi-class classification that yielded to an accuracy less than 90%.

### Detection of COVID-19 Through X-Ray Images

#### Binary Classification and Accuracy Less Than 90%

Hemdan et al. [70] developed COVIDX-Net framework for COVID-19 detection. This model used seven CNN architectures, including VGG19, DenseNet 201, Inception V3, ResNet V2, InceptionResNet V2, MobileNet V2, and Xception. VGG19 and DenseNet models achieved the best accuracy for COVID-19 classification. They yielded 0.89 and 0.91 F1-score for normal and COVID-19, respectively. The dataset consists of 50 X-ray images (25 normal images and 25 COVID images). Catak et al. [71] developed five different deep CNN techniques (VGG19, VGG16, ResNet, DenseNet, and InceptionV3) for identifying COVID-19 from X-ray images. The dataset contained 50 COVID-19 patients and 50 non-COVID-19 patients in the training phase; meanwhile, it contained 20 cases of COVID-19 and 20 cases of non-COVID-19 in the testing phase. VGG16 achieved the highest accuracy of 80%.

Horry et al. [72] developed pre-trained models (Xception, VGG16, VGG19, Inception v3, and RasNet50) to detect COVID-19 from X-ray images. VGG19 achieved the highest precision of 83%. The dataset contained 115 COVID-19 images. Haghanifar et al. [73] developed CheXNet model based on Xception, DenseNet, EfficientNet-B7, and ResNet for classifying X-Ray images into COVID-19, CAP, and normal. Their dataset contained 1326 COVID-19 images, 5000 normal images, and 4600 CAP images. The model achieved 87.88% accuracy.

#### Binary Classification and Accuracy More Than 90%

Ozturk et al. [74] developed an automatic detection model of COVID-19 from X-ray images. The proposed CNN consists of 17 convolution layers. This approach included both binary (COVID vs. no findings) and multi-class classification (COVID vs. no findings vs. pneumonia). The proposed approach used a darkNet model to classify You Only Look Once (YOLO) object detection system which is an algorithm used for detecting visual items in pictures. It achieved an accuracy of 98.08% and 87.02% for binary and multi-classes, respectively.Apostolopoulos & Mpesiana [75] proposed VGG19 for COVID-19 detection from chest X-ray images. VGG19 achieved an accuracy of 93.48 for binary classification and 98.75 for multi classification, respectively. The dataset contained 700 pneumonia, 504 normal, and 224 COVID-19 X-ray images.

Minaee et al. [76] developed DeepCOVID model based on 5071 X-ray images to distinguish COVID-19 from other lung pneumonia. The proposed model was trained through four CNNs: ResNet50, Dense-19, and SqueezeNet. In this model, a heatmap was generated to determine the regions infected by COVID-19. The evaluation of the model performance showed that SqueezeNet achieved the best performance. It reached 95.6% and 100% of specificity and sensitivity, respectively. Narin et al. [77] developed CNN models (ResNet50, ResNet101, ResNet152, Inceptionv3, and Inception ResNetv2) for three datasets. All datasets contained 341 COVID-19, dataset 1 contained 2800 normal, dataset 3 contained 2772 bacterial pneumonia which is caused by particular bacteria, and dataset 2 contained 1493 viral pneumonia chest X-ray images. The result showed that the ResNet50 has the highest accuracy, achieving 96.1%, 99.5%, and 99.7% for dataset1, dataset2, and dataset3, respectively.

Singh et al. [78] developed a detection model based on CNN and Multi-Objective Differential Evaluation (MODE) classifier which extract important information from the search data during evaluation process using clustering and statistical approaches, and then utilized to direct the production of new populations and local searches [79]. The model achieved 94.65% accuracy.

Pandit et al. [80] proposed a DCNN model for COVID-19 detection. The model used two datasets. The first contained 150 different patients of COVID-19, and the second was for collecting daily information about COVID-19 cases for statistical analysis. The proposed approach achieved an accuracy of 93%. Zhang et al. [81] developed a deep anomaly model for screening and detecting COVID-19 through X-ray images. The model was built on 100 images on 70 subjects confirmed as COVID-19 and 1431 images on 1008 subjects confirmed as another pneumonia. The result showed 96% and 70.65% accuracy for COVID-19 and non-COVID-19 cases, respectively. However, the study has limitations in missing 30% false positive rate and 4% of COVID-19 cases.

Alqudah et al. [82] developed COVID-19 identification model based on Support Vector Machine (SVM), Random Forest (RF), and CNN. Graphical features were extracted using CNN, and the difference between COVID and non-COVID images was performed using CNN classifier, RF, and SVM. The result showed that SVM was less time-consuming; however, in test stage, CNN achieved 95.2% accuracy.

Hossain el al. [83] applied ResNet 50 with ten different pre-trained weights on 7262 X-Ray images divided into COVID-19 and normal images. ResNet50 with iNat2021-Mini-SwAV-1K (iNMSwAV) achieved the highest score: 99.17%, 99.31%, and 99.03% for accuracy, precision, and sensitivity, respectively.

#### Multi Classification and Accuracy Less Than 90%

Khan et al. [84] proposed a CoroNet model COVID-19 detection through chest X-ray images. This model was pre-trained using ImageNet dataset [68]. CoroNet model achieved 89.6%, 93%, and 98.2% accuracy, precision, and recall, respectively, for four classes (COVID-19 vs. pneumonia viral vs. pneumonia bacterial vs. normal). The dataset of this model consists of 284 chest X-ray images for COVID-19, 310 for normal, 330 for pneumonia bacterial, and 327 for pneumonia viral.

Moutounet-Cartan [85] developed a Deep Convolution Neural Network (DCNN) model based on five CNNs (VGG19, VGG16, Xception, Inception v3, and InceptionResNetv2) to detect COVID-19. VGG16 achieved 84.1% accuracy. The dataset contained 327 X-ray images.

Pereira et al. [86] proposed a classification scheme to distinguish COVID-19 from other lung pneumonia. This model used a pre-trained CNN and resampling algorithms to balance the data. The proposed model achieved 89% F1-score for COVID-19 detection in hierarchical classification. This model used a database called RYDLS-20, containing 1144 chest X-ray images from 7 classes. In Nishio et al. [87] developed a Computer-Aided Diagnosis (CAD) system to distinguish between normal, COVID-19, and other pneumonia images. The developed models were based on the EfficientNet CNN and VGG16. These models combined data augmentation and Random Image Cropping and Patching (RICAP) techniques. The dataset contained 215 X-ray COVID-19 images, 500 normal images, and 533 other pneumonia. The model achieved sensitivity and accuracy of 90%, 83.6% sensitivity and accuracy, respectively.

Rahaman et al. [88] developed a CAD system for COVID-19 detection from X-ray images. VGG19 model achieved 89.3% and 0.90 accuracy and F1-score, respectively. This model contained 860 X-ray images.

Loey et al. [89] used deep transfer model techniques (GoogLeNet, ResNet18, AlexNet, and GAN to detect COVID-19 X-ray images. The dataset consisted of 306 X-ray images. They used three cases of the dataset. The first case consists of four groups of the dataset using GoogLeNet as the main technique for COVID-19 detection, achieving 80.6% testing accuracy. The second case consists of three groups of the dataset COVID-19, normal, and pneumonia bacterial groups. This case used AlexNet as the main transfer model and reached 85.2% accuracy. The third case consists of only two groups: COVID-19 and normal groups. The main transfer model was GoogLeNet and achieved 99.9% validation accuracy and 100% testing accuracy.

Monga et al. [90] applied six different transfer learning approaches: InceptionResNet V2, Xception, VGG19, VGG16, ResNet50 V2, and DenseNet201 for detecting COVID-19 from X-Ray images. Their dataset contained 770 chest X-Ray images divided into three classes: COVID-19, normal, and pneumonia. DenseNet201 achieved the highest performance with 82.8% accuracy.

#### Multi Classification and Accuracy More Than 90%

Wang et al. [91] implemented COVID-Net neural network for detecting COVID-19 from X-ray images. It was designed based on a combination of machine-driven design exploration and human-driven design principles. The used dataset is COVIDX, consisting of 16,756 chest images obtained from 13,645 patients. The model achieved accuracy and sensitivity of 92.4% and 80%, respectively.

Asif and Wenhui [92] implemented DCNN model built on inception v3 to detect COVID-19 from chest X-ray images. This model achieved 96.9% accuracy. There were 864 COVID-19, 1345 viral pneumonia, and 1341 normal chest X-ray images in the dataset. Narayan Das et al. [93] proposed the extreme version of inception to detect X-ray COVID-19 images. The dataset contained COVID-19, pneumonia but negative COVID-19, and other infections. The model reached 94.44% accuracy. Afshar et al. [94] developed a COVID-CAPS model based on a capsule network to detect COVID-19 in chest X-ray images. This model contained three capsule layers and four convolution layers. The proposed model achieved 95.7% and 90% accuracy and sensitivity in COVID-CAPS without pre-training, respectively. Meanwhile, the pre-trained COVID-CAPS achieved 98.3% and 80% accuracy and sensitivity, respectively.

Ucar and Korkmaz [95] developed a detection model based on deep Bayes-SqueezeNet. The dataset contained 1583 normal, 4290 pneumonia, and 76 COVID X-ray images. The model achieved 98.26% accuracy. However, Punn and Agarwal [96] developed DL models (InceptionResNet V2, ResNet, DenseNet169, Inception v3, and NASNetLarge) for COVID-19 detection. The dataset included108 X-ray images for COVID-19, 515 other pneumonia, and 453 normal images. NASNetLarge reached the highest accuracy for COVID-19 detection, achieving 98% and 99% accuracy and Area Under The Curve ( AUC), respectively.

Al-antari et al. [97] developed a CAD system that was built on the YOLO classifier to distinguish COVID-19 from other 8 diseases based on 50,490 images. It achieved 97.40% accuracy for classification. The study used two databases, chest X-ray8 in [98] and online COVID-19 database However, the system had some challenges, such as no availability for annotating images of digital X-rays and the need for physicians to label the COVID-19 lesion regions. The study’s future work is to make the proposed system to deal with CT images and use a Generative Adversarial Network (GAN) model for image synthesis. Narayanan et al. [99] developed a recommendation system to differentiate between COVID-19 and other several diseases, such as lung cancer, viral pneumonia, and bacterial pneumonia. The developed system was based on six different datasets and used four different CNN models (Inception v3, ResNet50, DenseNet 201, and Xception). The proposed approach used experiments of two hold-out validation and tenfold cross-validation. Ten folds cross-validation achieved 0.99 AUC for COVID-19 detection, whereas two hold-out validations achieved 0.94 sensitivity for COVID-19 detection.

Islam et al. [100] developed a COVID-19 detection model by combining CNN with Long Short-Term Memory (LSTM) which is a form of DL and has a feed forward connections. Features were extracted by CNN and LSTM used these features for classifying COVID-19. The proposed approach achieved a 98.9% F1-score. However, this approach has some limitations in that in a small sample size. It is unable to distinguish anterior-posterior where it solely concentrates on the posterior-anterior, COVID-19 images show various symptoms that are difficult to categorize. The dataset contained 4,575 X-ray images split into 3 groups: normal, COVID-19, and other pneumonia. Rahimzadeh and Attar [101] developed a combination model of ResNet50v2 and Xception to detect COVID-19 in X-ray images. This model used 2 datasets divided into 3 classes containing 180 COVID-19 images, 6054 pneumonia images, and 8851 images for normal cases. The proposed approach achieved 99.56% and 80.53% accuracy and recall, respectively. The accuracy of all classes was 91.41%.

Sethy et al. [102] implemented a combination model for COVID-19 detection. The model combined SVM with one of the 13 pre-trained CNN models: AlexNet, InceptionV3, DenseNet201, MobileNETv2, GoogLeNet, XceptionNet, InceptionResNETV2, ResNet18, ResNet101, SuffleNet, VGG16, ResNet50, and VGG19. The dataset contained 381 X-ray images; the combination of ResNet50 with SVM achieved the highest result by achieving 95.33% accuracy. Farooq and Hafeez [103] developed a COVID-ResNet50 model based on the ResNet50 technique to categorize X-ray images into four groups: COVID-19, healthy, bacterial pneumonia, and viral pneumonia. The dataset was COVIDX and it presented by COVID-Net researchers [91], consisting of 1203 normal images, 931 pneumonia bacterial images, 660 viral pneumonia images, and 68 COVID-19 images. The model achieved 96.2% accuracy in all classes of the COVIDX dataset and 100% accuracy for the COVID-19 class. Riahi et al. [104]developed a combination model of Bidimensional Empirical Mode Decomposition (BEMD) with 3DCNN. The initial X-ray images was decomposed to IMF by BEMD. They applied 3DCNN to the video created by IMFs to detect COVID-19. The dataset was obtained from [100], containing 1802 COVID X-ray images, 1910 normal images, and 272 viral pneumonia X-ray images. The developed model achieved 100% sensitivity and accuracy.

Moujahid et al. [105] applied Grade Cam technique and three different transfer learning approaches: VGG16, VGG19, and MobileNet V2 for classifying X-Ray images into pneumonia or normal or COVID-19. Their dataset contained 1341 normal images, 1345 pneumonia images, and 840 Covid-19 images. VGG19 achieved 96.97%, and 100% accuracy and F1-Score, respectively. ElGannour et al. [106]proposed two concatenation models. The first model is based on MobileNet V2, DenseNet 201, and ResNet50 V2. While the second model is based on Xception, Inception V3, and InceptionResNet V2 for classifying X-Ray images into Covid-19, normal, viral pneumonia, and tuberculosis. Their dataset contained 10,399 images. The models achieved 99.80% and 99.71% accuracy for the first and the second model, respectively.

#### Other Methods Related to Facing COVID-19 Spread

Maguolo and Nanni [107] reported the biases for classifying X-ray images for verifying the most suitable protocol for COVID-19 detection. Where most datasets came from almost the same sources. They used four different datasets and conducted different tests to know whether the classifier could determine the dataset source or not. They used AlexNet trained on images whose centers were black, and lung regions were deleted to detect COVID-19 images. Therefore, it would be impossible for the classifier to know anything about the source of the image or detection task. Results demonstrated that AlexNet could recognize COVID-19 images that came from the same or different sources without any biases and achieved 99.97% accuracy. The dataset contained 339,271 X-ray images. Similarly, Cohen et al. [108] studied the generalization performance of models for classification from chest X-ray images that came from the same or different sources. They used the DenseNet model for training on different datasets of A, B, C, and D. The result showed that if the model was trained on datasets B, C, and D and tested on A. The result would be less than if the model was trained on the B dataset and tested on B. With the publication of COVID-19 datasets by [109], they tried to merge the COVID-19 dataset to the chest X-ray dataset to classify these images and conduct testing on it. However, the study has limitations; it considers dataset labels only and does not consider patient outcomes. Boulila et al. [110] Proposed a new COVID-19 patient monitoring method that protects patient privacy in the setting of Saudi Arabia. It was a secure system for persistent patient monitoring thanks to the employment of inexpensive wireless devices and a cutting-edge encryption algorithm called chaos-based substitution boxes. To track daily activities and identify natural and unusual breathing rates. The system achieved 99% number of pixel change rate (NPCR).

Table 3 shows a comparison between these papers.

**Table 3:** Comparison between papers of X-Ray for COVID-19 detection

Paper	Model technique	Dataset	Evaluation measures	Limitations	Paper type	Publication date	Publication source
[84]	coroNet (deep convolution neural network)	284 COVID-19 images 310 normal images 330 bacterial pneumonia images 327 viral pneumonia images	89.6% accuracy 93% precision 98.2% recall	The model was built by a small dataset	Scientific paper	1 Nov 2020	Elsevier
[86]	Pre-trained CNN resampling algorithms	RYDLS-20(1144 images)	89% f1 score	The dataset was imbalanced: 1000 healthy images, 90 COVID-19 images, and the rest belonged to other diseases	Scientific paper	1 Oct 2020	Elsevier
[91]	COVID-Net (open source deep neural network)	COVIDX( 16,756 images)	92.4% accuracy 80% sensitivity	Model sensitivity of COVID-19 infections was very small	Scientific paper	11 Nov 2020	Nature Publication Group
[74]	darkNet	127 COVID-19 images 500 pneumonia images 500 non pneumonia images	98.08% binary classification accuracy 87.02% multi classification accuracy	The model was not validated by large number of images	Scientific paper	1 Jun 2020	Elsevier
[70]	COVIDX-NET, VGG16, DenseNet201 VGG19, MobileNet	50 images	91% f1 score	Dataset was very small, Accuracy of MobileNet should be improved more	Scientific paper	24 Mar 2020	Arxiv
[76]	DeepCOVID (ResNet50 DenseNet121 DenseNet18 squeezeNet )	5071 images	95.6% specificity 100% sensitivity	The dataset was imbalanced	Scientific paper	1 Oct 2020	Elsevier
[87]	CXDX system (Efficient Net, VGG16)	215 COVID-19 images 500 normal images 533 pneumonia images	83.6% accuracy 90% sensitivity	The model was validated only on public dataset which will be different from clinical data causing overfitting in external validation and CADX not be used by clinicians	Scientific paper	16 Oct 2020	Nature Publication Group
[75]	VGG19	700 pneumonia images 504 normal images 224 COVID-19 images	93.48% accuracy	More depth analysis requires for COVID-19 patients to detect mild symptoms that can not be visualized by X-Ray images	Scientific paper	Jun 2020	Springer
[85]	DCNN VGG19 VGG16 Xception Inception v3 InceptionResNet V2	125 COVID-19 images 152 normal images 50 pneumonia images	84.1% accuracy	The dataset was very small and imbalanced, It can not only be depended of the model for COVID-19 detection, professional diagnosis should be used beside	Scientific paper	2 May 2020	arXiv
[92]	DCNN and Inception v3	864 COVID-19 images 1345 viral pneumonia images 1341 normal images	96.9% accuracy	More deep learning approaches should be used for COVID-19 detection	Conference paper	11 Dec 2020	IEEE
[77]	ResNet50 ResNet 101 ResNet 152 Inception v3 Inception ResNet v2	2772 bacterial images 1493 viral pneumonia images 2800 normal images 341 COVID-19 images	96.1% accuracy DB1 99.5% accuracy DB2 99.7% accuracy DB3	Limited COVID-19 images	scientific paper	24 Aug 2021	Springer
[95]	Deep Bayes-squeezeNet	1583 normal images 4,290 pneumonia images 76 COVID-19 images	98.26% accuracy	COVID-19 images should be higher	scientif paper	July 2020	Elsevier
[71]	VGG16 VGG19 ResNet DenseNet Inception v3	70 COVID-19 images 70 non-COVID-19 images	80% accuracy	Small datasets size Model accuracy should be higher for COVID-19 detection	Scientific paper	1 Jan 2021	Elsevier
[72]	Xception Inception v3 ResNet50 VGG16 VGG19	115 COVID-19 images	83% precision	COVID-19 precision was so low and COVID-19 samples was very small	Scientific paper	21 April 2020	Engineering Archive
[96]	NESNetLarge Inception v3 DenseNet 169 ResNet ResNet v2	108 COVID-19 images 515 pneumonia images 453 normal images	98% accuracy 99% AUC	More deep learning and pre-processing techniques should be used for better performance, COVID-19 images was very small	Scientific paper	May 2021	Springer
[88]	CAD system depends on 15 different CNN techniques	860 images	90% f1 score 89.3% accuracy	More effective ensembles architectures should employed and Limited X-Ray images	Scientific paper	1 Jan 2020	IOS press
[97]	CAD system depends on YOLO predictor	50,490 images	97,40% accuracy	More help of physicians are needed for labeling the regions containing COVID-19 lesions	Scientific paper	May 2021	Springer
[80]	DCNN	150 COVID-19 patients	93% accuracy	Limited COVID-19 images	Scientific paper	1 May 2021	Elsevier
[81]	Deep anomaly model	100 images of COVID-19 1,431 other pneumonia images	96% accuracy	Small dataset size	Scientific paper	27 Nov 2020	IEEE
[100]	LSTM with CNN	1525 COVID-19 images 1525 pneumonia images 1525 normal images	98.9% F1 score	Small numbers of samples, the model focused only on posterior anterior (PA) view of images, it can not detect anterior posterior (AP) views in images, images with several COVID-19 symptoms can not be classified well,Model performance not compared with radiologists	Scientific paper	1 Jan 2020	Elsevier
[101]	CNN, ResNet50V2 Xception	6054 pneumonia images 8851 normal images 180 COVID-19 images	91.41% accuracy 80.53% recall	The model not well detected COVID-19 false class which made the model COVID-19 precision low, small number of COVID-19 images	Scientific paper	Jan 2020	Elsevier
[82]	CNN SVM Random forest		95.2% accuracy	Small dataset size, some other features like texture feature should be used for improving accuracy	Scientific paper	5 May 2020	JJEE
[89]	d GoogLeNet ResNET18 AlexNet GAN network	69 COVID-19 images, 79 normal images, 79 pneumonia bactrial images 79 pneumonia viral images	80.6% first scenario accuracy 85.2% second scenario accuracy 100% third scenario accuracy	Limited dataset size	Scientific paper	20 April 2020	MDPI
[102]	SVM classifier 13 transfer learning models	127 COVID-19 images 127 normal images 127 pneumonia images	95.33% accuracy	Limited dataset size	Scientific paper	2020	MDPI
[103]	COVID-ResNet50	COVIDx dataset (1203 normal images 931 pneumonia bacterial 660 viral pneumonia 68 COVID-19 images	96.2% accuracy	It can not be employed directly in clinical situations, the model needs more data to be more clinically useful	Scientific paper	31 Mar 2020	arXiv
[107]	AlexNet	339271 X-Ray images	99.97% accuracy	Limited COVID-19 images	Scientific paper	1 Dec 2021	Elsevier
[105]	VGG16 VGG19 MobileNet	1341 normal images 1345 pneumonia images 840 COVID-19 images	96.97% accuracy 100% F1-Score	Small number of COVID-19 images	Scientific paper	17 Nov 2021	Intelligent Automation and soft computing
[106]	Two concatenation models based on MobileNet V2, DenseNet 201, ResNet50 V2, Xception, Inception V3, InceptionResNet V2	10,399 images	99.80% first model accuracy 99.71% second model accuracy	Six publicly accessible datasets were taken into consideration in order to build a comparably balanced dataset due to the lack of coronavirus images in compared to the other diseases there was a significant increase in computational complexity and execution time	Scientific paper	29 Dec 2021	MDPI
[73]	CheXNet model based on Xception DenseNet EfficientNet-B7 ResNet	1326 COVID-19 images, 5000 normal images, and 4600 CAP images	87.88% accuracy	Ensemble models must be developed in order to improve the detection of thoracic abnormalities in CXRs utilizing CheXNet.individual sample variability brought on by changes in data ordering	Scientific paper	7 April 2022	Springer
[83]	ResNet50	7262 X-Ray images	99.17% accuracy 99.31% precision 99.03% sensitivity		Scientific paper	1 Jan 20222	Elsevier
[90]	InceptionResNet V2, Xception, VGG19, VGG16,ResNet50 V2, and DenseNet201	770 images	82.8% accuracy	Accuracy should be improved by applying more images dataset for COVID-19, bacterial, and viral pneumonia	book chapter	15 Mar 2022	Springer

### Detection of COVID-19 Through CT Scan Images

#### Binary Classification and Accuracy Less Than 90%

Shah et al. [111] created a CT-Net10 self-developed model for classifying CT scan images to COVID-19 or non-COVID-19 images. The developed model achieved 82.1% accuracy, reaching higher accuracy. CT-scan images were passed through multiple models (VGG16, ResNet50, Inception V3, DenseNet, and VGG19). VGG19 proved to be superior, achieving 95.52% accuracy. The dataset contained 738 CT-scan images and 349 COVID-19 images from 216 patients.

Shuai Wang et al. [112] developed a DL model to investigate the radiographic changes in CT images. The model used the adjusted inception transfer learning technique and was made on 1065 CT images. The model yielded 89.5% and 0.87 accuracy and sensitivity, respectively. However, it has challenges: poor signal-to-noise ratio and complicated data integration, which affected the efficiency of DL; the training dataset was small, which also affected the efficiency, and an enormous number of variable objects represented difficulty in the classification task. Therefore, their future work is to link the features of the clinical information and the genetic with the CT image hierarchical features to enhance diagnosis through multi-modeling analysis of these features.

Amyar et al. [113] developed a multitask model based on DL for detecting COVID-19 from chest CT images, determining disease severity through segmentation of the infected region from CT images, and making a reconstruction. The dataset came from multiple hospitals and contained 1369 CT images. These data are obtained from [114]. The model achieved 86% and 0.93 accuracy and AUC, respectively.Xiong et al. [115] applied an AI-based system to distinguish COVID-19 from other pneumonia. Chest CT image of the lung was first segmented by HU with a -320-thresholding value. Then, the segmented region was input to EfficientNet B4 deep neural network to classify COVID-19 and other pneumonia. The dataset contained 512 COVID-19 CT images and 665 non-COVID-19 pneumonia. The proposed model achieved 87% and 0.90 accuracy and AUC, respectively

#### Binary Classification and Accuracy More Than 90%

Wang et al. [116] developed (Decov Net) framework based on Unet and CNN to detect COVID-19 from CT images. Unet was used to segment the lung. Then, DNN used this segmented region to predict the infection probability of COVID-19. The model achieved 90.9% and 95.9% for accuracy and AUC, respectively. The dataset contained 219 and 313 non-COVID CT and COVID-19 images, respectively. However, it has some limitations; UNet trained with ground truth mask was imperfect, and the dataset came from an only single hospital, and there was no data for CAP in the study, where this was done, and the dataset of CAP that was added in [117].

Although Do and Vu [118] investigated several transfer learning models (VGG16, Xception, Inception V3, DenseNet201, Inception ResNet V2, DenseNet169, VGG19, and DenseNet21) for COVID-19 detection in CT scan images. The dataset contained 397 normal images and 349 COVID-19 images, respectively. DenseNet201 reached the highest accuracy in detecting COVID-19. It achieved 85% and 91% accuracy and recall, respectively. Their future work includes investigating a model for stacking several multiple architectures and integrating several imaging modalities into a single model.

Attallah et al. [119] developed a CAD system based on multiple CNNs to detect COVID-19 from CT images. The model employed different CNN techniques (GoogLeNet, AlexNet, ResNet18, and Shuffle-Net). The dataset contained 347 and 347 COVID and non-COVID CT images, respectively. It is available at [114]. The CAD system achieved 94.7% and 0.98 accuracy and AUC, respectively, which is better than [118]. However, it has some challenges: the need for a larger number of training data to differentiate COVID-19 from other pneumonia types and not supporting more segmentation techniques to distinguish between other tissues and the lung.

Gozes et al. [120] developed an automated analysis tool for tracking the progress of COVID-19 based on AI. The testing stage was conducted on 157 patients from China and the United States. The result showed that the developed system could extract lungs opacities slice automatically and produce a quantitative opacity measure as well as a 3D volume visualization for opacities. The experiment achieved 98.2% and 92.2% sensitivity and specificity, respectively.

Shan et al. [121] proposed a VB-Net neural network for quantification and segmentation of regions infected with COVID-19 and the entire lung from chest CT images. They aimed to evaluate disease progression and analyze changes in COVID-19 severity during the treatment period. They used 249 CT images of COVID-19 for the training phase and 300 COVID-19 CT images for validation and achieved a 91.6% Dice similarity coefficient between manual and automatic segmentation and yielded 0.3% mean Point Of Interest (POI) estimation error for the entire lung. However, it has some limitations; the dataset of validation was collected from one center, and this might not be represented all cases of COVID-19 from other areas. The proposed system only quantified infection of COVID-19 and did not quantify other pneumonia. Therefore, their future work is applying transfer learning to enable the system to quantify the severity of other pneumonia.

Chen et al. [122] developed a DL system to distinguish COVID-19 from other pneumonia. The proposed model employed UNet++ and a pre-trained ResNet50 on ImageNet dataset that is in [68]. The study was built on 20,886 CT images for COVID-19 from 51 patients and 14,469 CT images for other diseases from 55 patients. Due to a large number of images, the model achieved 100% sensitivity and 95.2% accuracy, which is higher than model accuracy in [119]. Jin et al. [123] proposed an AI system to detect COVID-19 through CT images and make a pipelined model that was built on ResNet50 and 3D Unet++. The dataset was collected from five different centers, which contained 723 and 413 COVID-19 and non-COVID images, respectively. It achieved 94.8% and 97.4% accuracy and sensitivity, respectively. Abbasian Ardakani et al. [124] developed a CAD system for COVID-19 diagnosis (COVIDag) based on 306 COVID-19 patients and 306 non-COVID patients. The proposed model used different classifiers (K-Nearest -Neighbor (KNN), Decision Tree (DT), Ensemble, SVM, and Naïve Bayes) to detect COVID-19 based on feature extraction of the image, lesion distribution, and ground-glass opacity. The ensemble classifier was the best among them, achieving 96.5% AUC. Their future work is to develop a model that can estimate the severity of the infected COVID-19 patient. Similarly, Afify et al. [125] developed a CAD system to detect COVID-19 based on 200 CT scan images, 100 images for COVID-19 and 100 for non-COVID obtained from [114], CAD system had five stages. The first stage was lung segmentation through threshold-based segmentation. Next, is the feature extraction on the segmented region, followed by feature selection performed using genetic algorithm. Then, they used the decision tree and KNN with *k* = 3 as a classifier of COVID-19. Finally, they obtained a performance analysis for the proposed model, in which KNN achieved 100% accuracy, whereas the decision tree achieved 95% accuracy.

Saeedi et al. [126] also developed a CAD system for online detection of COVID-19 from CT scan images. Users uploaded their images, and the system would give them the detection result. The proposed model was based on DenseNet 121 network for reduction of image dimensions and used NU-SVM to overcome over fitting problems. The proposed model also combined ResNet, MobileNet, and Inception. The developed approach achieved 90.80% and 90.61% recall and accuracy, respectively. The model was built on 349 and 397 COVID-19 and non-COVID-19 patients obtained from [114]. Ardakani et al. [127] created a CAD system based on ten pre-trained convolution layers (AlexNet, ResNet101, ResNet50, ResNet18, SqueezeNet, GoogLeNet, VGG16, VGG19, MobileNet-v2 and Xception) to classify COVID-19, and non-COVID-19 CT images. The dataset consisted of 1020 slice from 86 non-COVID-19 patients and 180 COVID-19 patients. The results showed that Xception and ResNet101 had the highest performance since both of them provided. 994 AUC. But, ResNet101 achieved 100% sensitivity while the sensitivity of Xception was 98.04%. ACAR et al. [128] developed a CAD system based on 7717 CT images to detect COVID-19 cases. The system used CT images with a Low Dose and CNN methods (LDCT )model to overcome of noise in low-dose CT images. ResNet 50 v2 was used for extracting features, quantum Fourier transform for lung segmentation, and t-SNE methods for determining the efficiency of features extraction. The developed approach calculated 99.5 %, 99% accuracy and sensitivity for detection of COVID-19, respectively.

Swapnarekha et al. [129] applied ResNet50 V2 and DenseNet201 for detecting Covid-19 from CT images. The used dataset contained 610 COVID-19 images and 600 non-COVID-19 images. ResNet50 V2 achieved 95.87%, 91.67%, and 100% of accuracy, specificity, and sensitivity, respectively. While DenseNet201 achieved 97.11%, 96.67%, and 97.54% accuracy, specificity, and sensitivity, respectively. Mete et al. [130] applied different deep learning approaches: VGG19, VGG16, AlexNet, Xception, GoogLeNet, ResNet50, SqueezeNet, and ResNet101 as a features extractors of 1345 CT images divided into COVID-19 and non-COVID-19 images. SVM, RF, DT, Naive Bayes( NB) and KNN were used as a classifier of these features. SVM and ResNet50 had the highest performance with 96.29%, 95.86%, and.9821 accuracy, F1-score, and AUC, respectively. Kogilavan et al. [131] proposed different deep learning models such as: Xception, DenseNet121, MobileNet, VGG16, NASNet, and EfficientNet for detecting COVID-19 from 3873 CT images. VGG16 achieved the best performance with 97.68% accuracy.

#### Multi-classification and Accuracy Less Than 90%

Xu et al. [132] developed an early screening model of COVID-19. The approach depended on several CNN models and Bayesian functions to detect COVID-19 and calculate the infection probability in CT images. The dataset contained 618 CT images. The developed approach achieved 86.7% accuracy for classifying COVID-19. Wang et al. [133] implemented a DL system for detecting COVID-19. The dataset contained 5372 patients. The mode achieved 0.87 AUC; however, it has limitations; not considering the prediction of events like admission to Intensive Care Unit (ICU), death, and distinct slice thicknesses of CT images are included in this study. Therefore, their future work is to convert CT images with different slice thicknesses into unified slice thicknesses of CT images using GAN.

Ying et al. [134] implemented a Deepneumonia model for identifying COVID-19. They developed a Detail Relation Extraction Neural Network (DRE-NET) model based on ResNet50 to extract the complex features from images. They also combined the pyramid network by attention module to classify COVID-19. The dataset consisted of 101 patients with bacterial pneumonia, 86 healthy patients, and 88 COVID-19 patients. The DRE-NET model achieved 86% and 95% accuracy and AUC, respectively.

Singh et al. [135] developed a DL model based on CNN and MODE for COVID-19 detection through CT images. The proposed model achieved superior accuracy than competitive models, such as Artificial Neural Network (ANN), CNN, and adaptive Neuro-Fuzzy Inference System (ANFIS) which merges the advantages of both ANN and Fuzzy Logic (FL) [136]. The result showed that the model could be used in real time to classify COVID-19 chest CT image from other pneumonia.

#### Multi-classification and Accuracy More Than 90%

Li et al. [117] developed COVNET framework, a Three-Dimensional (3D) DL based on ResNet50 to detect COVID-19, Community-Acquired Pneumonia (CAP) which is acute lung tissue infection in a patient who acquired it in the community or within 48 h of admission to the hospital [137], and other lung conditions through CT images. The dataset contained 4352 chest CT images. The proposed model achieved 0.96 AUC. However, it has limitations; it could not categorize the severity of COVID-19. Sharma et al. [138] proved the important role of machine learning techniques in fighting COVID-19 and knows whether CT scan image will be the first alternative reverse transcription-polymerase chain reaction (RT-PCR) in detecting COVID-19. Is COVID-19 different from any other pneumonia that resides on the lungs? How to distinguish between COVID-19 CT scan images and other kinds of lung CT scan images? To obtain all of this information, the authors employed customized software built on Microsoft Azure machine learning algorithms. The dataset contained 2200 CT scan images and the training model based on ResNet architecture and grad cam and achieved 91% accuracy.

Jin et al. [36] developed an AI-based system to classify CT images into four classes: COVID-19, CAP, influenza A and B, and non-pneumonia. The dataset was collected from different centers and contained 10,250 scans. The model achieved 97.17% AUC. However, it has some challenges. Guided grad cam did not achieve lesion segmentation, whereas if this was done, it would help phenotype analysis to work better in accurate segmented region. Their future work is to collect more CT images of other lung diseases to achieve higher performance. Zhang et al. [139] developed Novel Coronavirus Pneumonia (NCP) system for detecting COVID-19 based on chest CT images from 3777 patients. The model consists of two models: the lung lesion segmentation and diagnosis prediction models that took the segmented lung lesion from CT image as an input and classified it into COVID-19, or pneumonia, or normal. The proposed model achieved 0.97 AUC.

#### Other Methods Related to Facing COVID-19 Spread

Fang et al. [140] studied the travel history of two patients with COVID-19. The first patient was a 45-year-old woman, and another patient was a 32-year-old man to know whether CT images had the top sensitivity for detecting COVID-19 or RT-PCR. The result showed that CT images were the most effective in COVID-19 detection. Similarity, Xie et al. [141] compared RT-PCR and CT images, to know which one has the better accuracy in COVID-19 detection. The result showed that 3% of 167 patients had negative COVID-19 using RT-PCR, despite CT images showing that these patients had positive COVID-19. After some days, the result showed that CT images had better sensitivity for COVID-19 detection than RT-PCR. In addition, Bernheim et al. [142] studied the CT images of 121 cases from four different Chinese hospitals. They determined the relationship between symptom onset and CT scan and designated the signs of infection. The result showed that disease severity increased from the time of the first onset.

Zhang et al. [143] developed an analysis system for detection, quantification, and localization of COVID-19 out of chest CT images of 2460 patients. The proposed system could detect the infected region and measure the percentage of infection in the left and right lungs. However, the study has limitations; the intelligent assistant analysis system must be adjusted manually when identifying typical lesions. Singh et al. [144] proposed a lungINFseg model to determine the infected region of COVID-19 in CT images and make a lung segmentation for it. For estimation of the performance of lungINFseg, a comparative study was done between LungINFseg and other 13 different segmentation models (UNET, SegNet, SQNet,FCN,Inf-Net, ERFNET, ContextNet, FSSNet, DABNet, ESNet, CGNet, EDANet, and MISccn). LungINFseg achieved 80.34% dice score. The dataset contained 1800 annotated slice. The proposed model future work is making a good and accurate COVID-19 severity prediction by integration an automated CAD system with the proposed model. And applying the proposed model to another image segmentation problems like segmentation of breast tumor for ultrasound images.

Table 4 represents a comparison between these papers.

**Table 4:** Comparison between papers of CT images for COVID-19 detection

Paper	Model technique	Dataset	Evaluation measures	Limitations	Paper type	Publication date	Publication source
[117]	(COVNET) framework based on ResNet50	1296 COVID-19 CT exams 1735 CAP 1357 non pneumonia CT exams	96% AUC	Could’t categorize the severity of COVID-19	Scientific paper	19 Mar 2020	Radiology Society of North America
[111]	CT-net10 based on VGG16 ResNet50 InceptionV3 DenseNet VGG19 VGG19	738 CT-Scan images 349 images of them were COVID-19	95.52% accuracy	Small dataset size	Scientific paper	1 Feb 2021	Springer
[132]	Several CNNs models bayesian function	219 COVID-19 images 224 influenza A viral pneumonia images 157 healthy cases images	86.7% accuracy	There may be some overlap between COVID-19’s symptoms and those of other pneumonias. The study only used a small number of model samples, there should be more training and test samples available	Scientific paper	27 June 2020	Elsevier
[116]	(Decov Net) framework U-Net and CNN	219 non-COVID CT images, 313 COVID-19 images	90.9% accuracy 95.9% AUC	The U-net model could be enhanced by employing 3D segmentation networks and adopting exact ground-truth annotated by experts, Dataset came from only one hospital, Study did not include CAP dataset which would improve model performance, Due to the algorithm’s deep learning foundation and immature explain ability, it operated in a black-box manner when diagnosing COVID-19.	Scientific paper	20 May	2020 IEEE
[138]	ResNet Grad-Cam	800 COVID-19 images 600 viral pneumonia images 600 normal images	91% accuracy	There should be more numbers of model samples to improve model performance	Scientific paper	22 July 2020	Springer
[118]	VGG16 VGG19 Inception V3 Inception ResNetV2 Xception DenseNet201 DenseNet21 DenseNet169	397 normal images 349 COVID-19 images	85% accuracy 91% recall	Study contained limited CT images	Scientific book	21 Aug 2020	SPIE
[119]	CAD system based on GoogLeNet AlexNet ResNet18 shuffle-Net SVM as a classifier	347 COVID CT-images 347 non-COVID CT-images	94.7%accuracy 98% AUC	Small samples of COVID-19 images. Model only differentiated COVID-19 form non-COVID-19, not distinguish COVID from other pneumonia. The model performance didn’t compare with trained radiologists	Scientific paper	30 Sep 2020	PeerJ Inc
[133]	automated deep learning system	5372 CT images	87% AUC	The study didn’t consider death and ICU events. The study didn’t’ contain CT images with multiple slice thickness	Scientific paper	6 Aug 2020	European respiratory society
[120]	automated analysis tool based on AI	157 images	98.2% sensitivity 92.2% specificity	Limited dataset size	Scientific paper	24 Mar 2020	Arxiv
[121]	VB-Net neural network	279 COVID-9 CT images	3% mean POI 91.6% Dice similarity coefficient between manual and automatic segmentation	Limited dataset size	Scientific paper	30 Mar 2020	Arxiv
[112]	inception transfer learning technique	325 COVID-19 images 740 viral pneumonia images	89.5% accuracy 87% sensitivity	Small training samples. Model effectiveness has been hampered by a number of reasons, including low signal-to-noise ratio and difficult data integration. the characteristics of the CT scans were examined came from individuals with significant lung lesions at advanced illness stages.	Scientific paper	24 Feb 2021	Springer
[122]	UNet ++ ResNet-50	20,886 COVID-19 images 14,469 CT images for other diseases	95.2 %accuracy 100% sensitivity		Scientific paper	5 Nov 2020	Nature publishing Group
[113]	U-Net	425 normal images 495 other infection 449 COVID-19 images	86% accuracy 93% AUC	The study had a small samples number	Scientific paper	8 Oct 2020	Elsevier
[115]	EfficientNet B4 deep neural network	521 COVID-19 images 665 non-COVID-19 pneumonia	87% accuracy 90% AUC	Due to the radiologists in this study analysing the identical patients twice, first without and then with AI aid, there may be bias in results.The COVID-19 cohort’s distribution of the period between the beginning of symptoms and the CT was variable. The study limited sample size,Patients with COVID-19 pneumonitis had significant baseline differences from those with non-COVID-19 pneumonitis, which could have introduced bias.	Scientific paper	22 March 2021	Radiology society of North America
[36]	U-Net ResNet 152 Guided Grad Cam	11,356 CT	97.81% AUC	For improving diagnosis capabilities, gathering more data on more sub types of pneumonia or other lung diseases is beneficial.Guided Grad-CAM can only get the notice area rather than segmenting the lesion, and phenotype feature analysis would be better served by correct segmentation.	scientific paper	9 Oct 2020	Nature publishing Group
[123]	ResNet50 3D U-Net++	723 COVID-19 images 413 non-COVID images	94.8% accuracy 97.4% sensitivity	The study didn’t include dataset for other pneumonia	scientific paper	23 Mar2020	Cold Spring Harbor Press
[134]	Deepneumonia model, details relation extraction neural network (DRE-NET)model based on ResNet50	101 bacterial pneumonia patients 86 healthy patients 88 COVID-19 patients	86% accuracy 95% AUC	The model can’t resolve the batch effect to produce precise predictions for additional sources of data because the training data is still a tiny amount.	scientific paper	11 Mar 2021	IEEE
[139]	NCP system based on AI UNET DRUNET FCN SegNet Deeplabv3	3,777 patients	97% AUC 92.49% accuracy	The study contained small sample size	scientific paper	11 Jun 2020	Elsevier
[124]	CAD system (COVIDag) KNN, decision tree ensemble support vector machine naïve bayes as a classifier	306 COVID-19 patients 306 non-COVID patients	96.5% AUC	Preliminary RT- PCR’s results could provide erroneous negative results	scientific paper	31 Jan 2021	springer
[125]	CAD system genetic algorithm decision tree KNN as a classifier	100 images for COVID-19 100 images non-COVID	100% KNN accuracy 95% decision tree accuracy	Study contained small dataset size	scientific paper	25 Nov 2020	I IIETA
[126]	CAD system DenseNet 121 NU-SVM RasNet MobileNet Inception	349 COVID-19 patients 397 non-COVID patients	90.80% recall 90.61% accuracy	Small dataset size	scientific paper	24 Jun 2020	Arxiv
[127]	CAD system based on AlexNet ResNet101 ResNet50 ResNet18 SqueezeNet GoogLeNet VGG16 VGG19 MobileNet-v2 Xception	1,020 slice	99.4% ResNet_10 and Xception AUC 100% ResNet-101 sensitivity 98.04% Xception sensitivity	CAD evaluation and performance didn’t compare with radiologists. Negative RT-PCR results may be uncommon in COVID-19 patients	scientific paper	1 Jun 2020	Elsevier
[128]	ResNet50v2	7,717 CT images	99.5 % accuracy 99% sensitivity	Class imbalanced problem	scientific paper	4 Jan 2021	Cold Spring Laboratory press
[144]	lungINFseg model based on deep learning	1800 annotated slice	68.77% IOU 80.34% dice score	Study should integrate automated CAD system for provide better prediction of COVID-19 severity	scientific paper	22 Jan 2021	MDPI
[129]	ResNet50 V2 and DenseNet201	610 COVID-19 images, 600 non-COVID-19 images	96.67% DenseNet 201	Small dataset size	Conference paper	5 Sep 2021	Springer
[130]	VGG19, VGG16, AlexNet, Xception, GoogLeNet, ResNet50, SqueezeNet, and ResNet101 as a features extractors. SVM, RF, DT, Naïve Bayes( NB) and KNN were used as a classifiers	1345 CT images	SVM and ResNet50 had the highest performance with 96.29% accuracy, 95.86% F1-Score.9821 AUC	The study dataset was collected from only one hospital The dataset didn’t contain other different pneumonia	scientific paper	1 Sep	2022 Elsevier
[131]	Xception, DenseNet121, MobileNet, VGG16, NASNet, and EfficientNet	3873 CT images	97.68% VGG16 accuracy	Model couldn’t detect the COVID-19 affected areas	scientific paper	1 Feb 2022	Hindawi

### Detection of COVID-19 Through Ultrasound Images

#### Binary Classification and Accuracy Less Than 90%

Roy et al. [145] developed a model for automatic analysis of Lung US (LUS) images for COVID-19 detection. The model was built on a deep architecture and network to identify regions with pathological artifacts. This network could achieve localization of disease based on consistency losses. The proposed model could obtain an accurate COVID-19 diagnostic. However, it has some limitations: the small size of the dataset, the dataset collected from the same place, and the model needs a heterogeneous dataset to overcome model bias. Meanwhile, Karakuş et al. [146] proposed a method to quantify line artifacts of LUS images from 100 images of 9 patients with COVID-19. The model achieved 87% accuracy.

#### Multi-classification and Accuracy Less Than 90%

Born et al. [147] developed a DL framework to detect COVID-19 from ultrasound (US) images. They employed a POCUS dataset containing three classes of US images: 654, 172, and 277 images for COVID-19, healthy, and bacterial pneumonia, respectively. The developed framework (POCOVID-NET) was based on CNN and VGG16. The framework was evaluated in five fold cross-validation and achieved 89% and 96% accuracy and sensitivity, respectively.

#### Other Methods Related to Facing COVID-19 Spread

Moore and Gardiner [148] published a paper about the explanation of the importance of LUS images in detecting COVID-19, where US images could be used in ICU to identify lung conditions that might be required. The result showed that the LUS images are more sensitive than X-ray and CT images. In addition, US could be used to monitor different lung conditions, which help detect COVID-19 symptoms. khalili et al. [149] studied the importance of US images and the findings of COVID-19, such as pleural lines that are unsmooth and patchy consolidation. They discussed the advantages of LUS images. LUS could be used in ICU as an alternative for CT scan images. It has no radiation and has a lower cost; however, it has less sensitivity than CT images. Therefore, it could not be used for COVID-19 diagnosis since it has no ability for lesion detection. Table 5 represents a comparison between these papers

**Table 5:** Comparison between papers of US images for COVID-19 detection

Paper	Model technique	Dataset	Evaluation measures	Limitations	Paper type	Publication date	Publication source
[147]	POCOVID-NET CNN VGG16	POCUS dataset (654 COVID-19 images 172 healthy images 277 bacterial pneumonia images)	89% accuracy 96% sensitivity	Small dataset size	Scientific paper	25 April 2020	Arxiv
[145]	Deep architecture and network based on consistency losses and STN	277 LUS videos from 35 patients	70% precision 61% F1-Score 60% Recall	The study did not include heterogeneous and balanced dataset Patient demographics features were unknown. All datasets were collected from Italian hospitals and there was a bias in data collection, There were a noisy label in DS	Scientific paper	13 May 2020	IEEE
[146]	Function of nonconvex cauchy with method of line artifact quantification of ultrasound images	100 images from 9 COVID-19 patients	87% accuracy	A clinical drawback of LUS is that line artefact quantification relies on visual estimate, which may make it difficult to adequately depict overall fluid overload or the severity of illnesses, small dataset size	Scientific paper	6 May 2020	Arxiv

### Detection of COVID-19 Through Multi-model Imaging

#### Binary Classification and Accuracy Less Than 90%

Alom et al. [150] developed deep learning models for different tasks. The first model was for the classification of COVID-19 from X-ray and CT images. This model was built on Inception Recurrent Residual Neural Network (IRRNN). The second one was for segmenting infected regions in X-ray and CT images for detection and localization of COVID-19. The used dataset consisted of 420 X-ray samples, while the number of CT-Scan samples was 267. The model achieved 84.67 and 98.78% detection accuracy of COVID-19 for X-ray and CT-Scan, respectively. However, the main limitation of the developed model was the small sample size.

#### Binary Classification and Accuracy More Than 90%

Mukherjee et al. [41] developed a CNN-tailored Deep Neural Network (DNN) for the detection of COVID-19 from CT-Scan images and X-Ray. The model was built using two different datasets. The first dataset contained 168 COVID-19 X-ray images and 168 non-COVID images that included other diseases, such as SARS and MERS. The second dataset contained 168 COVID-19 CT-Scan images and 168 non-COVID-19 images. This model achieved 96.28% accuracy, 98.08% AUC, and 0.0208 Rate of False Positives. Jain et al. [151] applied transfer learning approaches such as: VGG16, MobileNet, Inception, DenseNet121, and ResNet50 for detecting COVID-19 and pneumonia diseases from X-Ray and CT images. VGG16 achieved the highest performance for X-Ray images with 99% accuracy. While DenseNet121 had the best performance for CT images with 97% accuracy.

#### Multi-classification and Accuracy Less Than 90%

Horry et al. [152] developed a framework for detecting COVID-19 from X-Ray, CT Scan, and ultrasound. VGG16 classified images into three classes normal, COVID, and pneumonia. The number of images in each class in the dataset is shown in Table 6. The ultrasound images had the best precision of 100%. Whereas, X-ray and CT achieved 86% and 84% precision, respectively.

**Table 6:** Dataset used in the study [152]

Image modality	Condition	Source images
X-Ray	COVID-19 pneumonia normal	140 322 60361
CT	COVID-19 non-COVID-19	349 397
US	COVID-19 pneumonia normal	399 277 235

#### Multi-classification and Accuracy More Than 90%

Panwar et al. [153] developed a transfer learning model for detecting COVID-19 through three different imaging datasets: pneumonia X-ray images, COVID-19 X-ray images, and SARS-COV-2 CT-Scan images. The developed VGG16-based model could detect COVID-19 faster than RT-PCR by 2 s. The experiments showed that there was a relation between pulmonary diseases, such as, COVID-19 and pneumonia. The model achieved 95.6% accuracy.

El Asnaoui and Chawki [154] developed an automated method to distinguish COVID-19 from normal and other pneumonia classes. The authors compared between different deep learning models, such as, DenseNET201, MobileNetv2, VGG16, VGG19, InceptionResNetv2, ResNet50, DenseNet201, and Inceptionv3. These models were developed using 6087 X-Ray and CT-Scan images. The result showed that InceptionResNetv2 had the best performance and it achieved an accuracy of 92.18%.

Gour et al. [155] proposed an ensemble model based on VGG19 and Xception for detecting COVID-19 from CT and X-Ray images. The X-Ray dataset contained 3040 chest X-Ray images divided into COVID-19, normal, and pneumonia images. The CT dataset contained 4645 images divided into COVID-19 and no-findings images. The proposed model achieved 97.62% multi-classification sensitivity for X-Ray images, and 98.31% binary-classification sensitivity for CT images.

#### Other Methods Related to Facing COVID-19 Spread

Sarosh et al. [156] developed a detection and segmentation model of COVID-19 from X-ray and CT-Scan images based on ResNet50, AlexNet and VGG16. The proposed model aimed to distinguish COVID-19 from other viral and bacterial pneumonia, CAP, and healthy images. This model aimed to identify and segment infected region in order to quantify the size and the ratio of infection. Similarity,

Table 7 shows a comparison between these papers

**Table 7:** Comparison between papers of multi-model images for COVID-19 detection

Paper	Model technique	Dataset	Evaluation measures	Limitations	Paper type	Publication date	Publication source
[152]	VGG16	140 X-Ray COVID-19 images 322 X-Ray pneumonia images 60361 X-Ray normal images 349 CT Scan COVID-19 images 397 CT Scan non-COVID images 399 Ultrasound COVID-19 images 277 Ultrasound pneumonia images 235 Ultrasound normal images	100% ultrasound precision 86% X-Ray precision 84% CT-Scan precision	The study contained small dataset size	Scientific paper	14 August 2020	IEEE
[150]	(IRRNN) Inception recurrent residual neural network. NABLA-N network	267 CT-Scan 420 X-Ray images	84.67 % X-Ray accuracy 98.78% CT-Scan accuracy	Class imbalanced problem, The CT model was tested by only 300 images, The COVID-Seg CT produces results with several false positive detection as a result of the lack of labelled data for lung segmentation in CT for COVID-19.	Scientific paper	7 April 2020	Arxiv
[153]	VGG 16 Grad-CAM	1252 COVID-19 CT-Scan images 1230 non-COVID- CT images 526 X-Ray images pneumonia 5856 X-Ray images for normal and pneumonia diseases	95.6% accuracy	Model should be tested with radiology images of patients with symptoms pneumonia	Scientific paper	22 Nov 2020	Elsevier
[41]	CNN-tailored DNN	672 images: 168 COVID-19 and non-COVID CT abd X-Ray images	96.28% accuracy 98.08% AUC	Small datasets size	Scientific paper	6 Nov 2020	Springer
[154]	DenseNET201 MobileNetv2 VGG16 VGG19 InceptionResNetv2 ReseNet50 DenseNet201 Inceptionv3	6087 X-Ray CT-Scan images ( 1,583 normal images 213 COVID-19 images 2780 bacterial pneumonia 1,493 corona virus images)	92.18% accuracy for InceptionResNetv2 88.09% accuracy for DenseNet201	The study had a small dataset, More techniques of feature extraction like YOLO should be used in the study for improving performance	Scientific paper	22 May 2020	Taylor and Francis
[155]	ensemble model based on VGG19 and Xception	3040 chest X-Ray images divided into COVID-19, normal, and pneumonia images. CT dataset contained 4,645 images divided into COVID-19 and no-findings images	97.62% multi-classification X-Ray sensitivity 98.31% binary classification CT sensitivity	The study should extend the model dataset for bacterial and viral pneumonia for achieving better performance	Scientific paper	1 Jan 2022	Elsevier
[151]	VGG16, MobileNet, Inception, DenseNet121, and ResNet50		VGG16 achieved the highest performance for X-Ray images with 99% accuracy. While DenseNet121 had the best performance for CT images with 97% accuracy.		book chapter	24 Jun 2022	IEEE

## Publicly Available Datasets

In the related work in Sect. 7, several datasets were cited. A summary of these datasets is provided in Table 8. This summary includes a reference to the dataset, its name, a brief description of the dataset, the type of images (X-Ray, CT, or US) included in this dataset, and the number of covid-19 samples.

The most frequently cited dataset is COVID-19 Image Data Collection [109]. This dataset was collected from different sources such as: Radiopaedia.Org [157], and Italian Society of Medical and Interventional Radiology (SIRM) COVID-19 Database [158]. This dataset provides academics working on artificial intelligence with COVID-19 images from several available publications and websites. Each image in this collection has a number of variables, such as: sex, date, age, survival, and medical records.

The COVID-19 Radiography Database [159], the leader of the COVID-19 Dataset Award, is a dataset that was created by combining data from different sources such as: [98, 109, 158, 160].

COVNET dataset [117], that contained 4352 chest CT was collected from 3,322 patients in six different hospitals in the period of August 2016 to February 2020.

The most widely used CT dataset is COVID CT Dataset [114]. This dataset contained 349 CT COVID-19 images with clinical findings of 216 COVID-19 patients and 463 non-COVID-19 images. It has meta data about patients such as: disease severity, age, gender, and his medical history. COVID CT Dataset was used in many studies such as: [119].

[138] A CT dataset that was collected from different sources such as: COVID CT Dataset, SIRM, and medical hospitals in Russia and India between 1 March 2020 to 25 April 2020.

In May 2021, an open access chest CT COVID-19 respiratory was released [161]. This dataset contained more than 1000 CT COVID-19 images collected from two hospitals of universities of Iran and Mashhad between March 2020 to January 2021. All images are 512 * 512 pixels stored in DICOM format.

The most used LUS dataset is POCUS dataset. It was published on May 2020 by Born et al. [147]. It contained 1103 images extracted from 64 videos divided into three groups: 654 COVID-19, 172 healthy, and 277 bacterial pneumonia. In 2021, a new version of POCUS dataset was released by Born et al. [162]. It is an updated POCUS dataset that contained 202 videos of COVID-19, healthy, bacterial, and viral pneumonia.

In March 2020 Soldati et al. [163] suggested a 4-level scoring system and a globally defined acquisition technique of LUS for COVID-19 patients. They revealed 30 COVID-19 positive instances in an online database, called ICLUS-DB, that contained over 60,000 frames. Roy et al. [145] updated this version of ICLU-DB to extend for 277 videos from 35 patients.

Finally, COVIDX-US dataset was released by Ebadi et al. [164] in March 2021. This dataset contained 12,943 frames from 150 videos. The images of the COVIDX-US dataset were divided into four classes: COVID-19, non-COVID-19, healthy, and other lung diseases.

It is important to mention that, various studies used different names for the same dataset. For example, some studies have referred to the COVID-19 Images Data Collection as the Montreal Data Base. Other datasets are not publicly available such as, the dataset used in [165].

**Table 8:** Summary of COVID-19 datasets used in the reviewed researches

References	Name	Brief description	Type	COVID-19 samples
[109]	COVID-19 Image Data Collection	Publicly available CXR COVID-19 with continuing update	X-Ray	315
[159]	COVID-19 Radiology Database	Publicly and ongoing updated dataset with more than 21,000 X-Ray images	X-Ray	3616
[91]	COVIDX dataset	Open access database with more than 13,975 CXR images	X-Ray	358
[84]	CoroNet dataset	Open access database containing 1251 CXR images belongs to 4 classes	X-Ray	284
[74]	X-Ray images dataset (DarkNet)	Publicly available CXR dataset belongs to three classes containing 1627 images	X-Ray	127
[98, 166]	Chest X-Ray 8 Chest X-Ray 14	Open access dataset which also called RSNA pneumonia detection challenge data or NIH CXR dataset, containing 108,948 frontal views CXR images	X-Ray	108,948 fontal views COVID-19 images
[157]	Radiopaedia. org	Open access website that enables to share radiography	X-Ray	20
[158]	SIRM COVID-19 Database	Publicly available dataset collected by Italian Society of Medical and Interventional Radiology (SIRM)	X-Ray	68
[167]	Augmented COVID-19 X-ray Images Dataset	Open access dataset containing 1,824 CXR images belongs to two classes	X-Ray	912
[160, 168, 169]	Labeled Optical Coherence Tomography (LOCT) Chest X-Ray Images (Pneumonia)	Open access chest X-ray images for classifying pneumonia belongs to three classes containing 5863 images	X-Ray	0
[114]	COVID-CT-Dataset	COVID-19 dataset containing 349 CT images from 219 patients	CT	349
[116]	deCOVnet dataset	Publicly dataset containing 2000 CT images belongs to two classes	CT	313
[117]	COVNET dataset	Open access dataset includes 4,352 CT images belongs to three classes	CT	1296
[170]	CORONACASES	Medical CT images for ten patients	CT	10
[171]	EURORAD.ORG	CT images for more than 10 COVID-19 patients	CT	10
[133]	Wangshuocas COVID-19	Open access dataset belongs to five classes containing 5372 CT images	CT	871
[161]	CT COVID-19 images	containing more than 1000 CT COVID-19 images	CT	1000
[147]	POCUS dataset	Open access and ongoing dataset with 64 videos resulting 1103 images divided into three classes	LUS	39 videos 654 images
[162]	Enlarged POCUS dataset	Updating version of POCUS dataset containing 202 videos belongs to four classes	LUS	
[163]	ICLUS-DB	LUS open access dataset includes 60,000 frames	LUS	30 cases
[145]	Extended ICLUS-DB	updated version of ICLU-DB to extend for 277 videos from 35 patients	LUS	17 cases
[164]	COVIDx-US	Publicly dataset containing 12,943 frames from 150 videos. divided into four classes	LUS	59 videos

## Discussion

Based on the data and conclusions presented in more than 100 articles explored. The findings of the primary search outlined in this paper are presented in this section. The following is a summary of these findings:

**Sub-RQ1: What are the main approaches for COVID-19 detection?**

As shown in Fig. 3, there are three primary methods for COVID-19 detection. These methods include blood tests, virus tests, and analyses of various imaging modalities like X-Rays, CT scans, and ultrasounds (US).The blood test is done to find out if there are any SARS-COV-2 antibodies present. The sensitivity of blood analysis, varies from 2% to 3% for the detection of SARS-COV-2. RDT and RT-PCR are the two methods used for the virus test. RDT is used to identify antibodies and can provide a speedy answer in around 30 min. It is not advised for COVID-19 detection, nevertheless, as its accuracy depends on the sample’s quality and it is unable to differentiate between COVID-19 and other viral pneumonia. RT-PCR is considered more reliable than RDT for detecting COVID-19, according to research [31]. But it has some restrictions. The process is expensive and time-consuming. Additionally, it has lower COVID detection sensitivity than imaging modalities, as its sensitivity ranges from 50 to 62% [33]. Some researches like [141] made a comparison between CT and RT-PCR to determine which method detects COVID-19 with the greatest degree of accuracy?. The research studied the travel history for 167 patients.Despite CT imaging indicating that these patients had positive COVID-19, the results of the RT-PCR test revealed that 3% of these patients had negative COVID-19. After a few days, the results revealed that RT-PCR was less sensitive than CT scans for the detection of COVID-19.

**Sub-RQ2: Which imaging modalities gives more accurate results? And what are advantages and disadvantages of each modality?**

The most effective technique to get quick and precise findings for COVID-19 detection is through imaging. The use of X-ray imaging for COVID-19 detection is encouraged because of their many benefits. These benefits include its greater accessibility and reduced cost compared to other imaging modalities. Additionally, X-ray image acquisition uses less radiation than CT scan image acquisition. As a result, it is utilized to identify several diseases, including lung cancer and cardiac conditions. The usage of X-ray images has become increasingly common, particularly in developing nations. On the other hand, the quality of CT scan images is superior than that of X-ray scans. As a result, the diagnosis outcomes from CT scan images are more accurate [35]. However, there are significant drawbacks of CT scans, including their high cost and the exposure of patients to more radiation. Regarding US images, Moore and Gardiner [148] and khalili et al. [149] discussed the importance and main advantages of US images. The results showed that the LUS images are lower sensitive than the CT and X-ray images for COVID -19 detection. However, since US uses no radiation and has a lower cost, it can be used in Intensive Care Units (ICUs) as a helper tool for tracking various lung problems.

**Sub-RQ3 How can COVID-19 be detected using AI and what AI tools are used in this detection?**

For detecting COVID-19 from X-Ray, CT, or US both machine learning and deep learning algorithms have been used. Some studies have used multiple machine learning algorithms such as, KNN, SVM, and DT for detecting COVID-19 either through X-Ray or CT like Abbasian Ardakani et al. [124] and Afify et al. [125]. Other studies have used transfer learning techniques such as, Xception, VGG, and Inception for providing better performance of Covid-19 detection like Catak et al. [71]. Many researches used various DL or ML classifiers to compare their performance in detecting COVID-19 like Jain et al. [151] and Abbasian Ardakani et al. [124]. Other researchers, Mete el al, [130] applied an ensemble method for detecting COVID-19 by using different DL algorithms, such as, VGG16, VGG19, ResNet 50, and Xception, as features extractors then fed these features to classical ML algorithms, such as, SVM, DT, and NB for classification. Gour et al. [155] proposed an ensemble model based on VGG19 and Xception for detecting COVID-19 from CT and X-Ray images by making both binary and multi-class classification. Researchers like Boulila et al. [110] applied AI technology in building a secure system for persistent patient monitoring thanks to the employment of inexpensive wireless devices and a cutting-edge encryption algorithm called chaos-based substitution boxes. To track daily activities and identify natural and unusual breathing rates. Tables 3, 4, 5, and 7 show the different AI techniques used for COVID-19 detection.

**Sub-RQ4 What are software tools and datasets used in building predictive COVID-19 detection model?**

The development tools that have been used in creating an AI detection COVID-19 models include the Matlab software and the python programming language. The python language has been the most common language for developing AI models. One reason is that python has a plenty of libraries such as, Numpy, Scikit-learn, TensorFlow, PyTorch, and Keras. Using these libraries eases the process of developing different ML and DL models, especially when using development environments such as, Google Colab or Kaggle notebooks.

To build efficient AI models, high quality datasets have to be available. Fortunately, multiple datasets of different imaging modalities that can be used to build such models are available. More details about these publicly-available datasets are provided in Sect. 8 and are summarized in Table 8.

**Sub-RQ5 How can DL provide a great weapon for fighting COVID-19 and what are the challenges it faced?**

Previous studies showed that DL techniques can provide great tools for detecting COVID-19. However, there are some challenges that can affect the accuracy of COVID-19 detection. The first challenge is the class imbalance problem that results from the limited size of COVID-19 images compared to other pneumonia and healthy images. To solve this problem, researchers like Rajaraman and Antani [172] used a data augmentation technique to increase the number of limited images by applying different transformations such as, translation and rotation on these images. Other researchers like Ucer and Korkmaz [95] used the SMOTE technique. There are other different techniques that can solve this problem like class-weighted entropy, cost-sensitive learning, and using an equal sample of each class. Researchers should choose the best solutions that fit their data.

The second challenge that DL models can face is the confidence of the model results; having high model results does not ensure having high certainty [173]. According to Ucer and Korkmaz [95], if the model produces results with a high level of uncertainty, it is recommended that human involvement should be used to further investigate the results. Ghoshal and Tucker [174], investigated Bayesian Convolution Neural Network (BCNN) for calculating the uncertainty in DL models. The developed DL models provided high or low level of the output certainty based on the COVID-19 X-ray input. The accuracy ranged from 86.02 to 89.82%. Therefore, the accuracy of prediction is significantly connected to the degree of uncertainty. In order to increase the level of trust in AI technology and to improve the process of disease diagnosis and treatment, more studies should consider the uncertainty problem in their models’ prediction.

The third challenge is sample overlap. The cause of this overlap is that many researchers have got their datasets from several online resources. As a result, the same image could be used several times in training and testing phases. One way to deal with this issue is performing image similarity analysis to figure out the images that are duplicated between the training and the testing datasets to remove this duplication. This will help in both reducing overfitting and preventing data sample overuse. Moreover, having COVID-19 datasets with main properties emphasized by radiologists will be more crucial for developing DL models. As the use of such datasets can improve DL models’ prediction and can be more acceptable by physicians in the diagnosis process.

The fourth challenge is disease seriousness. Analysis of COVID-19 images may aid in the identification of disease progression and the areas that require immediate assistance. These problems necessitate greater medical engagement at all the phases of development, evaluation, and validation of DL models. As in [175], the model could track the disease progression and predict from the extracted features whether the patient’s case would become worse or not.

As shown from the previous studies, most researches used transfer learning techniques for detecting COVID-19. Some studies used models pre-trained on ImageNet dataset, such as [84], and [122]. Others used models pre-trained on large dataset of images, such as [176]. Therefore, the selection of the suitable neural networks architectures for detecting COVID-19 should have more future research directions. Although many great efforts have been carried out for facing COVID-19 spread and detecting this disease, there are a number of future directions that should be done for providing better performance of COVID-19 detection. These main future directions include:

1: Building predictive models, beside the detection models, to predict whether individuals will be infected with COVID-19 or not based on their current locations, their current jobs, and the people who are contacting with them

2: Providing more accessible COVID-19 datasets with high quality images in order to develop models with better performance.

3: Most of the publicly-available Covid-19 datasets have small COVID-19 samples, therefore, theses datasets should be enlarged, to help researchers build more accurate detection models.

4: Many researches have used transfer learning techniques for developing COVID-19 detection models. Most of these models were pre-trained on ImageNet dataset, such as, [84], and [122]. While other models, such as, [176] and [83] were pre-trained on different datasets, such as, Chest X-ray 14, and iNat2021. Therefore, the selection of the suitable neural networks architectures for detecting COVID-19 should have more future research directions. [83].

## Conclusion

This research discussed a comprehensive survey about COVID-19 sources, its detection, its symptoms, and how AI can be used to stand against its spread. The paper discussed Coronaviruses’ families and their subgroups, COVID-19 sources, symptoms, and how it was transmitted from animals to human. This paper presented different DL approaches used in COVID-19 detection through different modalities, namely, X-Ray, CT, and US. The paper provided a comprehensive study about detecting COVID-19 from different approaches provided a comparison between them. It reviewed and compared between DL algorithms that can be used in COVID-19 detection, and highlighted their advantages and limitations in order to facilitate future developments in this area. It also highlighted the main features of each imaging modality in detecting COVID-19. Also, discussed the most frequently used datasets for COVID-19 and provided details about each dataset. Moreover, it showed that until today there is no accurate treatment for COVID-19. Therefore, future researches for COVID-19 detection should not stop to know all the details of this disease in order to help in fighting it.
